# Short chain fatty acids: key regulators of the local and systemic immune response in inflammatory diseases and infections

**DOI:** 10.1098/rsob.230014

**Published:** 2023-03-29

**Authors:** Lisa-Marie Ney, Maximilian Wipplinger, Martha Grossmann, Nicole Engert, Valentin D. Wegner, Alexander S. Mosig

**Affiliations:** ^1^ Institute of Biochemistry II, Jena University Hospital, Kastanienallee 1, 07747 Jena, Germany; ^2^ Center for Sepsis Control and Care, Jena University Hospital, Am Klinikum 1, 07747 Jena, Germany

**Keywords:** SCFA, immune metabolism, gut–liver axis, gut–lung axis, gut–brain axis, organ-on-chip

## Abstract

The human intestinal microbiome substantially affects human health and resistance to infections in its dynamic composition and varying release of microbial-derived metabolites. Short-chain fatty acids (SCFA) produced by commensal bacteria through fermentation of indigestible fibres are considered key regulators in orchestrating the host immune response to microbial colonization by regulating phagocytosis, chemokine and central signalling pathways of cell growth and apoptosis, thereby shaping the composition and functionality of the intestinal epithelial barrier. Although research of the last decades provided valuable insight into the pleiotropic functions of SCFAs and their capability to maintain human health, mechanistic details on how SCFAs act across different cell types and other organs are not fully understood. In this review, we provide an overview of the various functions of SCFAs in regulating cellular metabolism, emphasizing the orchestration of the immune response along the gut–brain, the gut–lung and the gut–liver axes. We discuss their potential pharmacological use in inflammatory diseases and infections and highlight new options of relevant human three-dimensional organ models to investigate and validate their biological functions in more detail.

## Graphical abstract

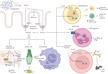

## 1. Introduction

The human microbiome consists of up to a thousand different bacteria, viruses, fungi and other protozoa found in the digestive system, skin, vaginal cavity and lungs. The influence of its composition and diversity on human health has become strikingly evident in recent years. Due to the complexity of how the microbiome communicates with the host at different levels ranging from single metabolites to microbial communities, identifying individual microbial-associated factors actively shaping human host response remains challenging. Among bacterial-derived metabolites, the short-chain fatty acids (SCFAs) acetate, propionate and butyrate belonged to the best-characterized molecules. They were proven essential mediators in the interaction between the human microbiota and its host [1].

The availability and composition of SCFAs are further influenced by nutrition. The macronutrient composition of the diet affects SCFA production, with a high in fat and protein diet resulting in reduced SCFA production, as gut bacteria preferentially ferment dietary fibre over fat. Conversely, a diet high in carbohydrates and low in fat can lead to an increase in SCFA production [2]. SCFAs are produced in the intestine by various bacteria such as Lactobacillus spp., Bifidobacterium spp., *Akkermansia muciniphila*, *Clostridium butyricum, Faecalibacterium prausnitzii* and others through saccharolytic fermentation of carbohydrates and proteins [1,3]. Conversely, dysbiosis or an imbalance in the gut microbiota can reduce SCFA production [4]. Soluble fibres such as pectin, beta-glucans and inulin are fermented more readily by gut bacteria than insoluble fibres such as cellulose and lignin [5]. As a result, consuming more soluble fibres is expected to lead to higher levels of SCFAs, particularly acetate, propionate and butyrate. Butyrate is produced in large amounts when gut bacteria ferment resistant starch, a type of carbohydrate that resists digestion in the small intestine. Consuming prebiotics such as fructooligosaccharides (FOS) and galactooligosaccharides (GOS) can also stimulate the growth of beneficial gut bacteria that produce SCFAs [5]. However, in a recently published systematic analysis, 12 studies were evaluated, reporting on the effects of dietary fibres on SCFA profiles [6]. Among those studies, seven studies demonstrated that the consumption of dietary fibres led to a significant increase in total SCFAs, while no significant changes were observed in the other five studies. The modulation of the SCFA profile was found to be highly influenced by the type of dietary fibre, its structure, and the dose that was consumed. In addition, the individual composition of the gut microbiota, which by itself is influenced by the body mass index (BMI), health/disease status and age [7], was also identified as a major factor affecting SCFA levels. Another layer of complexity to consider is the phenomenon of metabolic cross-feeding, defined as the interaction between bacterial strains in which metabolites resulting from the metabolism of one strain are further metabolized by another strain [8]. These effects might help to explain reports from studies where taxonomic changes were observed without a modification of the SCFA profile. Although the beneficial effects of dietary fibres are well established and widely accepted [9], it is still difficult to define how they affect the intestinal microbial ecosystem and SCFA levels in humans [6].

SCFAs are aliphatic acids absorbed by colonocytes and metabolized as substrates in the tricarboxylic acid (TCA) cycle [10]. The relative molar ratio of the SCFAs for acetate: propionate: butyrate in the human intestine is about 60: 20: 20, depending on the processed substrates [1]. From the intestine, SCFAs are distributed throughout the portal venous system to other organs, including the lung, the brain, the liver and the fat tissue [10–12]. As small molecules, SCFAs can diffuse through the cell membrane and act intracellularly as metabolic substrates in energy metabolism. Thereby, they are providing acetyl-CoA as the substrate for histone acetyltransferases (HATs) and are further potent inhibitors of histone-deacetylase complexes (HDACs), thereby regulating its target gene expression [13,14]. However, SCFAs can also directly bind and activate extracellular G protein-coupled receptors (GPCRs), which are free fatty acid receptor 2 (FFAR2), FFAR3 and hydroxycarboxylic acid receptor 2 (HCA2, GPR109A). These receptors have been demonstrated to play a significant role in SCFA-related signalling [15]. HCA2 is predominantly expressed by intestinal epithelial cells, adipose tissue and activated adipose tissue macrophages, and binds butyrate, but is not activated by propionate and acetate [16]. HCA2 plays an important role in suppressing colonic inflammation by promoting anti-inflammatory signalling and regulation of lipid homeostasis [17,18]. FFAR2 is mainly expressed in monocytes, eosinophils and neutrophils, regulating the chemotaxis of leucocytes and neutrophils [19]. In addition, FFAR2 is highly expressed by regulatory T cells (T_reg_) in the intestinal mucosa and regulates intestinal immune homeostasis [20]. In enteroendocrine L-cells, acetate and propionate induce the release of the glucagon-like peptide 1 (GLP1) upon SCFA-triggered elevation of intracellular Ca^2+^ levels via FFAR2-dependent signalling [21].

Furthermore, SCFAs play a profound role in regulating the immune response not only in the gut but also within the liver, the lung and in the central nervous system [22–24]. In this review, we will specifically highlight the pleiotropic functions of these small carbonic acids and their role in the metabolic crosstalk along the gut–lung, gut–liver and gut–brain axes, where they act as essential mediators of homeostasis and immune tolerance. We will further provide an overview of available *in vitro* model systems for exploring their therapeutic potential and characterizing their potential role in preventing or alleviating dysbiosis-associated diseases of the gut, the liver, the lung and the CNS.

## 2. Impact of SCFAs on peripheral immune cells

In recent years the emerging research field of immunometabolism was created by various studies that reported on the close interconnection between the metabolism and the immune response [25]. SCFAs have been demonstrated to affect innate immune cells such as macrophages, monocytes and neutrophils by rewiring their metabolism, inhibiting HDACs and suppressing signalling via nuclear factor-kappa B (NF-κB) [26,27].

### 2.1. T cells

The immunomodulatory effects of SCFAs on lymphocytes are well established. Indirectly, butyrate acts on T cells via macrophages and dendritic cells (DCs) in an HCA2-receptor depended manner on the regulation of T cell homeostasis and differentiation into naive and regulatory T cells (T_reg_). Butyrate modulates the release of IL10 and IL17, two central cytokines of the pathogenesis of inflammatory bowel disease (IBD) [17,28]. SCFAs were further shown to control the immune response of cytotoxic T lymphocytes (CTL) [29], and to increase the number of T_reg_ [28,30]. Acetate, propionate and butyrate can selectively support the development of T helper cells (Th) 1 and Th17 effector cells and IL-10 secreting T_reg_ depending on the cytokine milieu and the immunological context. These regulatory pathways are independent of FFAR3 and FFAR2 signalling but rely on the inhibition of HDACs and increased mTOR–S6K activity [31]. The activation of CD8^+^ T lymphocyte activity involves the increase of the mitochondrial volume with SCFAs directly acting as a substrate for fatty acid oxidation (FAO) and the generation of acetyl-CoA, which fuels the tricarboxylic acid (TCA) cycle and oxidative phosphorylation (OXPHOS) [32]. This mechanism has been found relevant to promote CD8^+^ T cell long-term survival as memory cells. CD8^+^ T cells require priming by professional antigen-presenting cells to participate in the immune response against intracellular pathogens and tumours. Bachem *et al*. described microbiota-derived butyrate to promote the memory potential of antigen-activated CD8^+^ T cells [33]. In the study, butyrate promoted CD8^+^ T cell long-term survival by shifting the cellular metabolism through uncoupling the TCA cycle from glycolysis and promoting OXPHOS by utilizing glutamine and FAO. Studies on the tumour responsiveness to chemo- or immunotherapies revealed that butyrate could also boost the efficacy of the chemotherapeutic drug oxaliplatin by modulating CD8^+^ T cell function and promoting the IL-12 signalling pathway in the tumour microenvironment [34]. In adoptive T cell therapy (ATC), pre-treatment of cytotoxic CAR T cells with SCFAs enhanced antigen-specific anti-tumour activity. In CTLs, pentanoate and butyrate increased the function of mTOR, a central cellular metabolic sensor that induces metabolic alterations associated with cytolytic activity against targeted tumour cells [35]. Pentanoate caused an anti-inflammatory phenotype characterized by increased release of IL-10 and downregulation of IL-17A production in CD4^+^ T cells. Like butyrate, pentanoate inhibits HDAC function but further acts as a substrate for HATs after its metabolization to acetyl-CoA [36]. In the same study, pentanoate was also demonstrated to protect from autoimmune pathology by inducing the formation of regulatory B-cells in experimental mouse models of colitis and multiple sclerosis [36].

### 2.2. B cells

Both butyrate and propionate have recently been demonstrated to reduce local and systemic antibody responses in a dose-dependent manner modulating epigenetic imprint in B cells. Mechanistically, both SCFAs act as HDCA inhibitors and downregulate activation-induced cytidine deaminase (AID) and B lymphocyte-induced maturation protein-1 (Blimp1) expression, thereby inhibiting class-switch DNA recombination, somatic hypermutation and plasma cell differentiation in C57BL/J6 mice. Butyrate and propionate upregulated miRNA expression that target transcripts and silences genes of activation-induced cytidine deaminase (Aicda) and PrDM (which encodes B lymphocyte-induced maturation protein-1 (BLIMP-1)), thereby inducing the impairment of intestinal and systemic T-dependent as well as T-independent antibody responses [37]. However, in another study, SCFAs induced the differentiation of B-cells into plasma cells and stimulated class switching with increased release of IgA by fuelling the Acetyl-CoA pool [38]. By contrast to T cells, this metabolic switch relied on the expression of FFAR2 [39].

Activation of B cells and the release of inflammation-promoting antibodies plays a central role in the onset and progression of rheumatoid arthritis (RA). Recently changes in the gut microbiota and alterations in the formation of SCFAs were found to alleviate disease symptoms in animal disease models and RA patients [40]. In mice, regulatory B cells (B_reg_), immunosuppressive cells that contribute to maintaining immunological tolerance [41], were shown to limit inflammation in RA. The serotonin-derived metabolite hydroxy indole-3-acetic acid (5-HIAA) was found to mediate the activation of B_reg_ via the transcriptional regulator arylhydrocarbon receptor (Ahr), which supported B_reg_ function and thereby alleviated the severity of RA.

The regulation of an appropriate B cell response is crucial in the intestinal mucosa to protect against human pathogens and their toxins. Cholera toxin (CT), an enterotoxin secreted by *Vibrio cholerae*, is a potent adjuvant for inducing mucosal immune responses. Depleting the gut microbiota with antibiotics decreased both systemic and mucosal antibody responses induced by CT. In line with this observation, oral supplementation of mice with acetate or butyrate restored IgA and IgG antibody responses to CT in an FFAR2-dependent manner [42].

### 2.3. Macrophages

Macrophages can adopt various complex and transient activation patterns depending on their specific microenvironment. For simplicity, these activation patterns are often termed M1 and M2 polarization stages. Although this simple concept neglects essential aspects of the complete biological complexity of macrophage plasticity, it still helps mechanistic studies identify and characterize regulatory pathways controlling inflammatory processes. The M1 polarization is characterized by a proinflammatory profile, increased glycolysis and a disrupted TCA cycle resulting in the accumulation of succinate and itaconate [43]. By contrast, M2-polarized macrophages, which are involved in tissue remodelling, show an anti-inflammatory profile and rely on using the TCA cycle and OXPHOS as primary energy sources [44,45]. In the intestine, tissue-resident macrophages form a protective barrier against invading pathogenic microorganisms. They are replenished primarily by blood monocytes and act as critical gatekeepers shaped in their activation pattern by commensal and pathogenic microorganisms [46,47]. SCFAs produced by the gut microbiota are essential regulators of macrophage polarization. Butyrate has been shown to shift the metabolism of M2 macrophages towards OXPHOS and FAO [48]. In vitro, butyrate decreases glycolysis resulting in higher AMP levels, activation of AMP kinase and the suppression of mTOR kinase activity. This metabolic shift is associated with an improved light chain-3 (LC3) -mediated phagocytosis and increased antimicrobial peptide synthesis [49]. Consequently, the antimicrobial potential of butyrate-treated macrophages is boosted by butyrate and could be counteracted by stimulation of mTOR [46].

### 2.4. Neutrophils

Neutrophils belong to the most abundant cell types of the peripheral immune system and provide a first-line immune defence by phagocytosing, killing and digesting bacteria and fungi. Neutrophils generate energy from glucose's metabolization via glycolysis [29,50]. In neutrophils, SCFAs have been shown to directly act on FFAR2, thereby increasing their migration to sites of inflammation [16]. Acetate has been found to enhance innate immune responses of neutrophils via FFAR2 and to promote the activation of the inflammasome, with subsequent release of IL-1*β*. This antibacterial host response is supported and coordinated with innate lymphoid cells type 3 (ILC3s) that augment IL-1 receptor expression in an FFAR2-depended manner upon acetate binding and secrete IL-22 in response to IL-1*β* stimulation [51]. In line with this observation, the loss of FFAR2 reduces the number of recruited neutrophils by acetate or butyrate in the intestine [52].

Increased neutrophile recruitment by butyrate is responsible for its antimicrobial activity in *Clostridioides difficile* (*C. diff*.) infections (CDI). In a CDI mouse model, the colonization with the butyrate-producing bacterium *Clostridium butyricum* (CBM588) consequently ameliorated inflammatory disease symptoms associated with increased neutrophil invasion and elevated Th1 and Th17 cell counts during the early phase of CDI [53]. SCFAs were further found to specifically inhibit the generation of nitric oxide (NO) in neutrophils [54]. However, in the context of inflammatory bowel disease, butyrate suppressed *in vitro* neutrophil migration and the formation of NETs in cells from patients suffering from Crohn's disease and ulcerative colitis. This was validated in a DSS-induced colitis model of mice, where butyrate significantly ameliorated mucosal inflammation by inhibiting NET formation and the release of proinflammatory cytokines, chemokines and calprotectin [55].

These findings underline the importance of specific environmental cues affecting SCFA-related signalling pathways and their provoked functions.

## 3. SCFAs regulate organ-specific functionality

### 3.1. Gut

Acting as an immunocompetent barrier, the gut regulates not only the uptake of nutrients and electrolytes but is also essential for preventing the translocation of pathogens and their toxins into the bloodstream. The mucosal immune system is key in fine-tuning the complex mutual interaction with the microbiota, thereby discriminating between beneficial commensals and opportunistic pathogens [56]. As part of the microbiome, SCFAs mediate an adapted intestinal immune response toward a dynamically changing microbiota [57]. In the intestine, SCFAs can be passively taken up through diffusion by intestinal epithelial cells or via the sodium-coupled monocarboxylate transporter1 (SMCT-1) and butyrate via monocarboxylate transporter 1 (MCT-1) [58]. Butyrate is the primary energy source for colonocytes providing 60–70% of their energy supply [59]. Butyrate further promotes the transcription of tight junction proteins such as Claudin-1, stabilizing epithelial integrity and improving intestinal barrier functionality [59]. Under ‘physiological hypoxia’, the oxygen consumption in enterocytes is driven by butyrate, which stabilizes the hypoxia-inducible factor 1 (HIF-1), a transcription factor that upregulates gene expression of Claudin-1 [60,61].

Butyrate further stimulates the synthesis of antimicrobial peptides (AMP), thereby shaping intestinal microbiota composition [62]. Consequently, prolonged antibiotic treatment could disturb intestinal homeostasis and induces dysbiosis by decreasing colonization with SCFA-producing commensal bacteria [60]. The mucus layer secreted by intestinal goblet cells provides a substrate for colonizing bacteria and acts as a barrier that separates the microbiota from direct interaction with epithelial cells. Butyrate can improve mucin production by increasing the frequency of mucin-secreting goblet cells in the colon crypts in a macrophage-dependent manner. Mechanistically, butyrate facilitates M2 macrophage polarization while blockade of Wnt secretion or ERK1/2 activation suppresses the beneficial effect of butyrate-primed macrophages on goblet cell function. In line with this, the adoptive transfer of butyrate-induced M2 macrophages in a dextran sulfate sodium (DSS)-induced mice model of colitis restores mucus secretion. It ameliorates disease symptoms by stimulating goblet cell regeneration [63]. In clinical studies, the oral administration of sodium butyrate as a nutritional supplement was further shown to induce the secretion of the antimicrobial peptide AMP cathelicidin (CAMP), REGIII*β*/*γ* and β- defensins by human intestinal epithelial cells [62,64]. In addition to butyrate, acetate and propionate directly modulate the epithelial immune response (i.e. by inducing the release of the pro-inflammatory cytokine IL-18, which is proposed to improve intestinal barrier function [65]). Further, IL-18 belongs to key epithelial-derived cytokines regulating intestinal CD4^+^ T cell subsets and contributes to maintaining intestinal immune homeostasis [66].

SCFAs mount anti-inflammatory conditions in the intestine by activating HCA2, expressed by intestinal dendritic cells and macrophages, and mediate the release of anti-inflammatory cytokine IL-10, which subsequently promotes differentiation of T_reg_ [17,18,67,68]. T_reg_ expressing the transcriptional regulator FoxP3 are critical in regulating intestinal inflammation, with their differentiation depending on the microbial colonization of the mucosa. The number of Treg in the colon is significantly decreased in germ-free mice. Still, it could be reconstituted to physiological levels by oral administration of spore-forming SCFA-producing *Clostridium* bacteria [30,69]. T_reg_ differentiation is stimulated by the inhibition of HDACs through SCFAs promoting histone H3 acetylation of the Foxp3 gene promoter [30]. The mechanism was recapitulated *in vivo* by applying the HDAC inhibitor trichostatin A, which induces the expansion of Foxp3^+^ T_reg_ and attenuated colitis in mice [70].

SCFAs are also central regulators in preventing IBD, a chronic illness linked to the dysregulation of the mucosal immune response to luminal antigens [71,72]. The disturbance of the composition of the intestinal microbiome is typically seen in IBD. It might be directly linked to an unfavourable composition of SCFAs, which can regulate the release of anti-microbial peptides. In IBD, the main subtypes ulcerative colitis and Crohn's disease can be distinguished. Several lines of evidence from *in vitro* and *in vivo* studies demonstrated the beneficial effect of SCFAs as a potential treatment option for both subtypes of IBD. In animal studies, oral administration of butyrate ameliorates mucosal inflammation and prevents gut barrier impairment [73]. Also, propionate was shown to enhance T_reg_ function in an FFAR2-dependent manner, protecting mice from colitis [74]. This has been validated in FFAR2-deficient mice that show an exacerbated or unresolved inflammation in experimentally induced colitis [75]. Results from clinical trials support this finding for ulcerative colitis patients receiving pre- and/or probiotic treatments to augment the endogenous formation of SCFAs [76–78]. However, the beneficial effect of SCFAs for Crohn's disease patients through supplementation of SCFAs or oral uptake of pre- or probiotics to alleviate disease symptoms is less clear [77].

**Figure 1.**
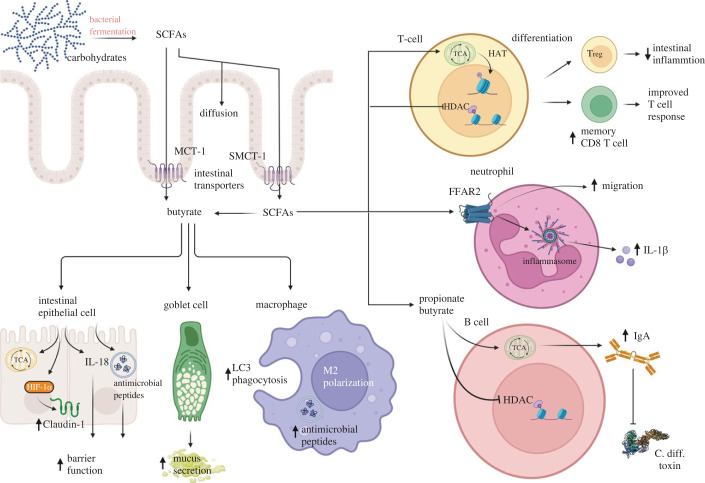
SCFAs are central modulators of the mucosal immune response. They are produced by fermenting commensal bacteria in the intestine and could passively diffuse or be actively transported by MCT-1 or SMCT-1 through the intestinal barrier. SCFAs could act as an energy source for intestinal epithelial cells, contribute to maintaining intestinal barrier function by upregulating Claudin-1 expression, stimulate mucus secretion and induce M2 macrophage polarization with increased LC3-phagocytosis to protect from invading pathogens. Their effects on peripheral immune cells include stimulation of Treg differentiation, boosting neutrophil migration to sites of inflammation, and induction of B cell antibody class switch and antibody secretion against toxins (i.e. from *C*. *diff*.). Created with BioRender.com.

Dysbiosis is frequently observed as a significant side effect of prolonged antibiotic treatment, which is also an important predisposing factor for *C. diff*. infections, causing intestinal diseases ranging from mild diarrhea to pseudomembranous colitis. In addition to its function on neutrophils, oral supplementation of sodium butyrate was shown to protect from *C. diff.*-induced colitis by reinstalling physiological hypoxic conditions and stabilizing HIF-1*α* expression in intestinal epithelial cells [60,61]. A summary of the different functions of SCFAs on intestinal epithelial cells, and peripheral and tissue-resident immune cells within the intestine is provided in figure 1.

Impairment of the intestinal barrier is also linked to the onset of cystic fibrosis [79,80], allergic asthma [81], non-alcoholic fatty liver disease (NAFLD) [82,83] and multiple sclerosis (MS) [84]. In the following sections, we will discuss the role of intestinal-derived SCFAs as regulators of the inflammatory response in the liver, the lung and the central nervous system (CNS) and the potential therapeutic options of SCFA supplementation in ameliorating disease-associated inflammation in these organs.

### 3.2. Liver

The liver is the central organ for metabolizing nutrients and drugs absorbed by the intestine. SCFAs produced in the gut are shuttled to the liver via the bloodstream of the portal venous system [1]. By contrast to acetate, butyrate and propionate are almost entirely eliminated from the bloodstream by the liver [12]. In western countries, NAFLD represents an increasingly relevant disease, affecting a quarter of the USA and Asian population [85]. The disease is characterized by lipid accumulation in hepatocytes and increased cell death. It can develop into non-alcoholic steatohepatitis (NASH), which ultimately results in liver cirrhosis with a higher risk of end-stage liver diseases, such as liver decompensation and hepatocellular carcinoma. Accumulating data indicate the association between dysbiosis, low levels of SCFAs and the onset of NAFLD [86]. SCFAs are able to reduce lipid deposition in the liver by stimulating AMP-activated protein kinase (AMPK) and inducing the expression of FAO in a peroxisome proliferator-activated receptor-γ (PPAR*γ*)-depended manner [87,88]. Further, acetate suppresses proinflammatory activation of liver macrophages, thereby limiting hepatic inflammation and alleviating NASH symptoms [87]. Beneficial effects of SCFAs have been reported for butyrate, which protects against the onset of insulin resistance and NAFLD by inhibiting NF-kB activation through limiting Toll-like receptor (TLR) activity in the liver [89]. Experiments in rats revealed a significant improvement in liver dysfunction after partial hepatic ischemia upon treatment with butyrate. The improvement was associated with limited nuclear translocation of NF-κB p65 and a decreased release of the pro-inflammatory cytokines TNF*α* and IL-6 by Kupffer cells [90]. In mice, butyrate protects against western-style diet-induced NASH by reducing TLR-4-dependent inflammation and improving the metabolism of lipids and glucose [91]. In line with this, the colonization of mice with 12 different strains of the commensal SCFA-producing bacterium *Faecalibacterium prausnitzii* significantly restored serum lipid profiles and ameliorated glucose intolerance, adipose tissue dysfunction, hepatic steatosis, inflammation and oxidative stress in a NAFLD disease model [92].

Acetate fuels cellular energy metabolism and represents a building block in cholesterol synthesis [10,93]. Still, studies demonstrated a significant decline in serum cholesterol levels induced by acetate and propionate by decreasing the enzyme activities in the cholesterol synthesis cycle [88,94]. Further, intestinal cholesterol uptake is reduced by propionate through inhibition of the major cholesterol transporter Niemann-Pick C1-like 1 (Npc1l1) [95].

The progression of NAFLD to liver cirrhosis is a major risk factor for developing hepatocellular carcinoma. Hepatocellular carcinoma formation was found to be associated with dysbiosis and decreased levels of acetate-producing bacteria resulting in diminished IL-17A secretion by hepatic ILC3 [96]. Several studies have reported anti-tumorigenic effects of SCFAs on cancerogenesis [96–98]. Butyrate could inhibit tumour growth by suppressing HDAC activity and inducing apoptosis in cancer cells by modulating the miR-22/SIRT pathway resulting in the upregulation of ROS production, the release of cytochrome c, and the activation of caspase-3 [99]. In vivo, the administration of butyrate during chemotherapy allowed a reduction in the concentration of the chemotherapeutic drug irinotecan [100] and reduced the toxic side effects of irinotecan in rats [101]. However, there are also reports that increased SCFA concentrations in the portal venous blood could create a tumour-promoting microenvironment for hepatocytes with expansion of T_reg_ and attenuation of CD8^+^ T cells [102,103].

### 3.3. Lung

The lung has long been considered a sterile organ that, under healthy conditions, is not colonized by bacteria, viruses, or other microorganisms. However, with the rise of culture-independent tools for detecting microbes in recent years, there is now evidence that also in the lungs, at least a transient microbiome exists under homeostatic conditions [104]. It is assumed that respiratory colonization occurs primarily via the micro-aspiration of microorganisms from the oropharynx but is also influenced by the microbiome composition of the upper gastrointestinal tract [105]. Similarly, the diversity in the lung microbiome decreases with age and with the severity of diseases [106]. In healthy subjects, the composition of the microbiome in the lower respiratory tract is indistinguishable from microbial colonization in the supraglottic respiratory tract. The composition of this microbiome has typically been influenced by the microbial immigration within the respiratory tract, the elimination of microorganisms by the immune system, and the relative microbial reproduction rates [107]. The development of lung diseases such as cystic fibrosis and chronic obstructive pulmonary disease (COPD) is associated with a shift in the composition of the lung microbiome [108], which is reflected at SCFA levels in the lung [109]. Vice versa, IBD patients show an increased prevalence of COPD, indicating disease-relevant crosstalk between gut and lung via SCFAs [110,111]. However, it is still unclear whether the observed changes in the lung microbiome composition are causative of impaired lung function or simply reflect new local growing conditions related to the disease progression.

The connection between the gut and lung is already formed during embryonic development, where booth organs derive from the common embryological origin of the primitive foregut [112]. Although SCFAs are detectable in human lungs [113] the required substrates for SCFA formation through fermenting bacteria are missing indicating that SCFAs found in the lung originate from the gut. The presence of various SCFA receptors such as olfactory receptor-78 (OLFR78) in the upper airways, and FFAR3 and FFAR2 in alveolar macrophages, alveolar type 2 progenitor cells, airway smooth muscle cells and the airway epithelium, however, suggests that SCFAs have a functional role in the lung as well [113–115]. Clear indication for functional crosstalk between gut and lung involving acetate is provided by the observation of newborns that have a significantly increased risk of developing bronchial asthma and atopy by the age of 3 years when they develop an intestinal dysbiosis with reduced acetate levels already at the age of three months [116]. The disease pattern of bronchial asthma is characterized by airway hyperreactivity (AHR) and inflammation driven by a deviating Th2 response. In this context, a high fibre-associated incline in SCFA production in the gut was demonstrated to prevent the progression of AHR [117]. In mice, butyrate-producing gut bacteria suppress inflammation in AHR by modulating oxidative phosphorylation and glycolytic metabolic pathways of pulmonary ILC2. In this study, butyrate was demonstrated to downregulate GATA3 expression resulting in reduced eosinophilic inflammation and less mucus formation [118]. Thio *et al.* were also able to validate the butyrate-mediated reduction of proinflammatory cytokine production release in human ILC2 cells underlining the potential of butyrate supplementation for the treatment of AHR [119]. Butyrate, propionate, and partially acetate have been shown to have beneficial effects on the treatment of asthma. Huang *et al.* investigated the influence of SCFAs on the inflammatory process in an ovalbumin-induced asthma model of mice [120]. The authors studied alternatively activated M2 macrophages that can promote the production of Th2 cytokines, accumulate inflammatory cells, and stimulate mucus secretion in AHR. In this context, the adoptive transfer of M2 macrophages enhanced airway inflammation in *Aspergillus fumigatus*-induced asthma.

By contrast to reports from the gut and liver, butyrate, propionate and acetate inhibited M2 macrophage polarization in an FFAR2-dependent manner and were found to be protective against the development of airway inflammation and AHR by inhibiting HDAC activity [120,121]. There is further evidence that the lung microbiome of diseased patients responds differently to SCFAs [109]. In a model of cystic fibrosis, bronchial epithelial cells from patients expressed higher levels of FFAR3. They showed no decrease in IL-8 secretion even at very high propionate concentrations compared to epithelial cells derived from healthy donors [109].

Intestinal colonization with SCFA-producing bacteria was further shown to mediate protective effects in pneumonia, where infection with *Klebsiella pneumoniae* was resolved faster after *Bifidobacterium longum* (*B. longum*) administration. The colonization with *B. longum* led to the release of anti-inflammatory IL-10 in the lung, protecting infected animals from severe tissue damage and significantly reducing their mortality [122]. Hagihara *et al*. reported important crosstalk along the gut–lung axis after oral administration of the butyrate-producing bacterium *Clostridium butyricum,* which increases the resistance to influenza virus infection after upregulation of interferon (IFN)-λ in lung epithelial cells*.* The ω-3 fatty acid 18-hydroxy eicosapentaenoic acid (18-HEPE) enhanced ω-3 fatty acid sensitivity in the lungs by promoting the expression of GPR120 and induction of IFN-λ release [123]. However, in another study, high-dose propionate treatment of the lung reduced the immune containment in *Staphylococcus aureus* pneumonia and diminished the host defence against the pathogen [124]. This illustrates the importance of carefully considering the complex modulation of the inflammatory response by SCFA regarding their local and systemic availability to unfold its host protective effects but to avoid any detrimental dose-dependent immunosuppressive effects in the course of infections.

### 3.4. Central nervous system

Intestinal barrier perturbation and the resulting inflammatory conditions are associated with the development of various neurological diseases [125,126]. The communication between the CNS and the intestine with its enteric nervous system (ENS) is mainly mediated by the sympathetic and parasympathetic nervous system and various hormones regulating gut mobility and appetite [127,128]. There is evidence of bidirectional crosstalk between the CNS and the intestine [129]. SCFAs directly impact physiological processes on the blood-brain-barrier (BBB) integrity and the cellular energy metabolism along the gut–brain axis. Butyrate is capable of preventing the translocation of inflammatory agents through the BBB by improving its integrity [130,131].

Furthermore, acetate has been found to play an important role in the energy metabolism of the CNS by increasing acetyl-CoA- and ATP levels in the brain [132,133]. Acetate also represents an important energy source for astrocytes [134]. Both butyrate and acetate further dampen the inflammatory response in lipopolysaccharides (LPS) induced neuroinflammation [135,136] and are required for the maintenance of homeostasis and the maturation of microglia cells by modulating neuroinflammation in an FFAR2-dependent manner [137].

Propionate seems to have an important function in MS [84] where its level in the intestine is significantly decreased [138]. A causal link for propionate has been demonstrated in a mice model of induced experimental autoimmune encephalitis (EAE), an animal surrogate of MS. In this model, the neurological symptoms associated with EAE were ameliorated by administration of propionate [139]. These results were confirmed by a clinical study with MS patients, where the standard drug medication was amended with propionate supplementation over three years. A *post-hoc* analysis revealed a significant reduction in relapses and brain atrophy and normalization of T_reg_ function in propionate-supplemented MS patients [138]. Like MS, also the treatment of Parkinson's disease and Alzheimer's disease was found to be improved by the supplementation with SCFAs highlighting their attractive therapeutic potential through amelioration of the disease-associated neuroinflammatory processes [140,141]. In a *Drosophila* model of Parkinson's disease, butyrate application improved locomotor impairment and elevated dopamine levels resulting in higher survival rates [142]. These observations are supported by studies with murine and human neuronal cells in which the neuroprotective effect of butyrate was also confirmed [143–145]. In mice models of Alzheimer's disease, butyrate improved memory function in the advanced stage of the disease [146], inhibited aggregation of beta-amyloid [147] and reduced neuroinflammation that improved synaptic plasticity [148]. Furthermore, butyrate ameliorated the social behaviour in autism spectrum disorder (ASD) through the improved expression of apical junctional complex proteins at the BBB, thereby limiting neuroinflammation and disease progression [125,149].

In a murine stress model with stress-induced intestinal damage, the oral admission of SCFAs resulted in lowered corticosterone release and decreased anxiety-like and depressive-like behaviour [150,151]. Further, butyrate induced the expression of genes linked to an anti-stress response by inhibiting HDAC activity [152–154]. Its anti-depressive effects are related to reduced microglia cell activation and diminished TNF*α* signalling [155–157]. Based on the results of these studies, the supplementation of butyrate represents a promising treatment option for depression and other stress-related diseases.

**Figure 2.**
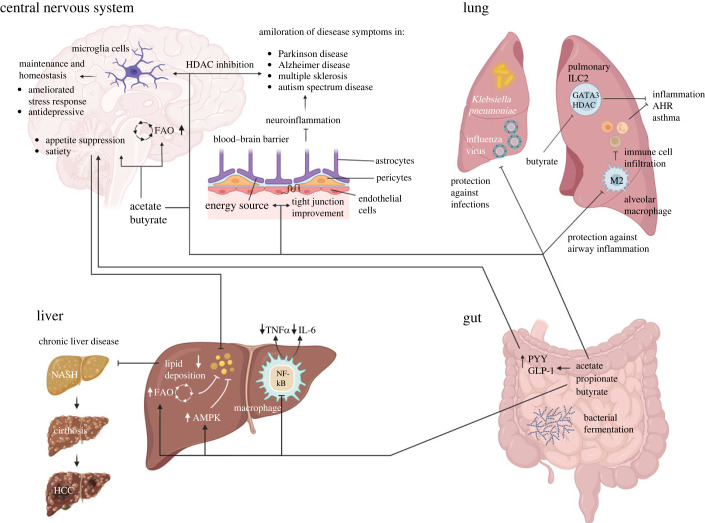
SCFAs produced and released from the gut are distributed via the bloodstream, eventually reaching the liver, the lung and the CNS. SCFAs can be metabolized via FAO or in the TCA cycle. They can mediate anti-inflammatory effects, contribute to tissue homeostasis, protect from blood barrier breakdown, and alleviate symptoms of inflammatory diseases and neural disorders. HCC, hepatocellular carcinoma; AHR, airway hyperreactivity; NASH, non-alcoholic steatohepatitis. Created with BioRender.com.

Acetate was reported as a modulator in the release of the hypothalamic neuropeptide [158]. Interestingly, acetate can even reduce liver steatosis by suppressing appetite by acting on enteroendocrine cells and glutamate-glutamine and GABA neuroglial cycles associated with increasing hypothalamic lactate [159]. Mechanistically, FFAR2 and FFAR3 expressed by enteroendocrine cells mediate the release of peptide YY and GLP-1 [160], both important mediators of satiety [161,162]. In another study, butyrate supplementation was associated with reduced food uptake, promoted FAO and activation of brown adipose tissue with increased utilization of plasma triglyceride-derived fatty acids, which prevented diet-induced obesity, hyperinsulinemia, hypertriglyceridemia and hepatic steatosis [163]. The most important pleiotropic functions of SCFAs in the crosstalk between the gut and the lung, the CNS and the liver are presented in figure 2.

## 4. State-of-the-art models to study the effects of SCFAs

Most existing studies on SCFA function have used animal models, while clinical studies are scarce. Rodents are well-established animal models for studying the systemic effects of SCFAs and their multiple functions on various organs [20,95]. In particular germ-free mice were used in a multitude of studies to explore the role of the microbiome and how it is shaping the human host's immune response. However, the translational potential of rodent models to the human situation is limited due to alterations in the immune system of germ-free mice and more general species-related differences in the function and composition of the immune system between animals and men [164–167]. Given the vast pleiotropic effects of SCFAs in different organs and individual cell types in a complex mutual interaction of the microbiota with its host, it is challenging to avoid technical bias in the complex animal organism. Although animals provide a ‘complete picture’ in terms of biological complexity, reductionistic but scalable *in vitro* models offer an attractive option to systematically address mechanistic questions of SCFAs from the cellular to the tissue level under defined and controllable experimental conditions in human genetic background.

Current *in vitro* models often use cancerogenic cell lines, which show important alterations in SCFA metabolism compared to primary cells. In cancer cells, SCFAs could accumulate due to the Warburg effect, which describes the uncoupling of aerobic glycolysis from oxidative respiration. Under these conditions, intracellular butyrate and propionate levels incline faster due to their limited metabolization. They could inhibit HDAC activity more efficiently with altered histone acetylation compared to non-cancerogenic cells. Another issue in standard cell culture models to consider is the influence of glucose, which is in standard cell culture media for immortalized cell lines and is often present in very high non-physiological concentrations. Under low, physiologically more relevant glucose conditions, the oxidative metabolism in cancer cells is increased, and butyrate and propionate are metabolized more efficiently, thereby lowering their epigenome-modulating effects [168]. As most of those *in vitro* studies were performed with cancer cell lines, these mechanisms should be considered in the extrapolation of *in vitro* SCFA effects to the *in vivo* situation [168].

An interesting alternative to cancer cell lines are organoids that have become more and more important as sophisticated *in vitro* models to study the human host-microbiome interaction. The cellular arrangement in these tissue models allows the execution of developmental programs capable of specifically guiding intricate, temporally varying signalling dynamics of stem cells for determining its cell fate and cell linage commitment in a spatio-temporal manner to form an organotypic structure that recapitulates essential microanatomical features with high cellular diversity [169,170]. Cell growth and differentiation of the stem cells are guided by genetically preserved embryonic developmental programs enabling these organoids to recreate cellular polarization and to mimic a realistic expression pattern of receptors and transporter proteins for SCFAs. In such organoid models, it has recently been shown that the crypt architecture formed by colonocytes protects the stem cell niche through a metabolic barrier by consuming butyrate, thereby preventing the suppression of intestinal stem cell proliferation by butyrate at the base of the crypts [171]. Intestinal organoids derived from patients with colorectal cancer (CRC) were further used to study the effects of SCFAs on the responsiveness to radiotherapy. By contrast to propionate and acetate, only butyrate was able to suppress the proliferation of organoids and enhance radiation-induced cell death. Importantly, radiation-induced cell death was not enhanced in normal organoids, where it improved the regeneration capacity after irradiation [172].

However, organoids tend to vary in their morphology, size and number depending on donor-specific characteristics and culture conditions. Further, a standardized and reproducible coculture of a living microbiota inside organoids is challenging, as the number of bacteria and volume needed for microinjection depends on the individual organoid size and lumen, which tends to be heterogenous among donors and even within one batch from a given donor. The size and morphological variability of organoids in standard cultures are further impracticable for standardization in studies when larger sample numbers are required. Organ-on-chip models offer a way to circumvent some of the limitations of conventional organoid cultures. In most of the models, a porous membrane serves as a functionable cell culture substrate this is separating two channels and supporting cell growth by providing a scaffold for perfusion with cell culture medium and removal of cellular waste products [173–177].

**Figure 3.**
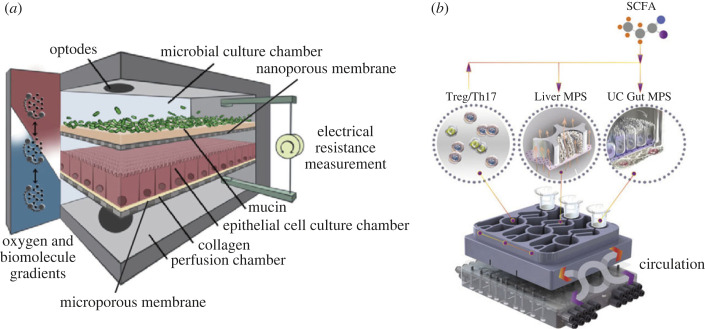
Organ-on-chip models for studying SCFA function in the gut and along the gut–liver axis. (*a*) Annotated schematic illustration of the key features of the HuMiX platform. A microporous membrane serves as a cell culture substrate for epithelial cells that can be perfused in the chip. A nanoporous membrane separates a living microbiota cocultured in the chip from direct interaction with epithelial cells but allows diffusion of microbiota-derived metabolites. The cell culture system allows the generation of oxygen gradients to mimic physiologically relevant culture conditions. Optodes are used to monitor the oxygen saturation of the perfused cell culture medium. Integrated electrodes measure transepithelial electrical resistance (TEER) to quantify the permeability of the epithelial cell layer modulated by microbiota-derived metabolites (with permission from Elsevier, [178]). (*b*) Schematic illustration of a human microphysiological system integrating organ models of the gut, the liver, and circulating T_reg_ and Th17 cells. In the multi-organ model, disease conditions of ulcerative colitis (UC) were simulated. SCFAs were shown to improve or worsen UC severity, depending on the involvement of effector CD4^+^ T cells (with permission from Elsevier [179]).

Gut-on-chip models were used to study the effects of SCFAs on the barrier function and regulation of the immune response *in vitro*. The HuMix model allows the coculture of a living microbiota with intestinal epithelial cells and immune cells under physiologically relevant anaerobic conditions (figure 3*a*). In this model, the commensal bacterium *Lactobacillus rhamnosus* GG (LGG) was cocultured with human intestinal epithelial cells and induced similar transcriptional, metabolic and immunological responses as observed *in vivo* [180]. The HuMix system was further used to coculture colon rectal cancer (CRC)-derived epithelial cells with a probiotic and prebiotic treatment. Using multi-omics and in silico metabolic modelling, it was demonstrated that the combination of probiotic and prebiotic stimuli caused distinct ratios of SCFAs and induced the downregulation of genes involved in pro-carcinogenic pathways and drug resistance. Formate, an SCFA produced by *Fusobacterium nucleatum*, has been identified in a follow-up study in the HuMix model to promote CRC development [181]. Patient-derived CRC cells were cocultured with *F. nucleatum* and displayed a metabolic shift toward increased formate secretion. The authors could show the formate-mediated protumorigenic effects by driving CRC tumour invasion through triggering AhR signalling and increasing cancer stemness. These findings were validated in mice where *F. nucleatum and* formate treatment caused an increased tumour incidence and tumour size associated with an expansion of Th17 cells favouring a proinflammatory milieu.

The modulation of T cell responses by SCFAs has also been studied in a multi-organ system emulating the gut–liver axis in the context of ulcerative colitis (figure 3*b*). Conditions of inflammation were recreated in the gut model by coculture of primary human UC epithelial cells with dendritic cells and macrophages. In this model, the exposure to SCFAs decreased innate immune cell activation through PPAR signalling and the downregulation of the NF-kB pathway. The gut model was coupled to a model of the liver, in which the hepatic metabolic function improved due to the reduced inflammatory profile in the gut system. In the multi-organ model, the conversion of SCFAs was increased and resulted in enhanced production of bile acids and increased gluconeogenesis, lipid metabolism, and the formation of ketone bodies. Surprisingly, SCFAs induced increased hepatocyte damage by acting on T cells and stimulating metabolic reprogramming toward increased differentiation of circulated CD4^+^ T cells into activated T_reg_ and Th17 cells through inhibiting HDAC and p62-TRAF6 function [179].

Several groups have established *in vitro* models of the BBB [182–184] and even more complex models of the neurovascular unit (NVU), which also includes glia cells or neurons [185,186]. With these systems, advances have been made in modelling specific neurological disorders, such as Alzheimer's disease [187,188] and Parkinson's disease [189–191]. In future studies, these model systems will help to investigate SCFAs and their role in modulating signalling processes and the immune response along the gut–brain axis. New modelling approaches with multi-organ-on-chip systems will further contribute to the evaluation of the therapeutic potential of SCFAs for the treatment of neurological and neurovascular disorders.

## 5. Conclusion

SCFAs are potent modulators of the host immune response and efficiently orchestrate the crosstalk between the intestine and the lung, the liver and the CNS. Its specific effects are mainly a result of induced metabolic and metagenomic changes by SCFAs and FFAR-related signalling on both the adaptive and the innate immune system. Recent data from various studies highlight the pharmacological potential of SCFAs in acute and chronic inflammatory diseases and infections. However, still many questions remain open. To fully explore the pleiotropic effects of SCFAs and to understand the multiple signalling processes related to SCFA binding, uptake and metabolization, a reasonable combination of emerging three-dimensional organ models and well-characterized animal models will provide more insight into SCFA biology and its therapeutic potential. Still, it remains challenging to define the therapeutic potential of SCFA (i.e. on the improvement of IBD or in the context of carcinogenesis). Although SCFAs show apparent effects in reducing the inflammatory response in sterile or allergic transmitted lung injuries, their therapeutic use for treating reparatory diseases still needs further evaluation. Thus, more extensive clinical trials are required to unravel the pharmaceutical potential of SCFAs in diseases such as Crohn's disease, ulcerative colitis, NASH, hepatocellular carcinoma or pneumonia.

## Data Availability

This article has no additional data.
